# Prevalence of Metabolic Syndrome in an Adult Urban Population of the West of Iran

**DOI:** 10.1155/2009/136501

**Published:** 2009-11-04

**Authors:** F. Sharifi, S. N. Mousavinasab, M. Saeini, M. Dinmohammadi

**Affiliations:** ^1^Clinical Endocrinology, Metabolic Diseases Research Center, Zanjan University of Medical Sciences, Zanjan, Iran; ^2^Statistical Sciences, Metabolic Diseases Research Center, Zanjan University of Medical Sciences, Zanjan, Iran; ^3^Non Communicable Diseases Prevention Center, Zanjan University of Medical Sciences, Zanjan, Iran; ^4^Department of Health and Prevention, Zanjan University of Medical Sciences, Zanjan, Iran

## Abstract

*Objectives*. We determine the prevalence of the metabolic syndrome in an urban population of Zanjan, a province located to the west of Tehran. *Methods*. Randomly selected adults >20 years were studied using stratified sampling. Target study sample was 2941 (1396 males and 1545 females). Metabolic syndrome was diagnosed using Adult Treatment Panel-III (ATP-III) guidelines when any three of the following were present: central obesity, raised triglycerides ≥150 mg/dl, low high-density lipoprotein (HDL) cholesterol, blood pressure ≥ 130/ ≥ 85 mm Hg, and diabetes or fasting plasma glucose (FPG) ≥ 100 mg/dl. *Results*. Metabolic syndrome was present in 697 (23.7%) subjects (CI 95%:22%–25%, *P* = .001), prevalence was 23.1% in men and 24.4% in women (*P* : .4). The prevalence increased from 7.5% in the population younger than 30 y to 45.6% in ages more than 50 years. Low HDL was the most common metabolic abnormality in both sexes. Most of those with metabolic syndrome had three components of the syndrome (75.6%), 170 subjects (24.4%) had four and none had five components simultaneously. The prevalence of obesity (BMI ≥ 30 kg/m^2^), hypercholesterolemia (≥200 mg/dl) and high LDL cholesterol (≥130 mg/dl) was greater in the metabolic syndrome group than normal subjects (*P* = .00). *Conclusions*. There is a high prevalence of metabolic syndrome in this urban population of the northern west of Iran. Focus of cardiovascular prevention should be undertaken in this area.

## 1. Introduction

The name of metabolic syndrome has been considered for the clustering of some cardiovascular risk factors from 1988 [1]. Because of the different criteria for the definition of the syndrome, the prevalence of the metabolic syndrome has varied markedly between different studies [2, 3]. This syndrome has become common in the United States [4] and there are some reports about its increasing prevalence in Asia [5]. Although some studies have reported the prevalence of individual cardiovascular risk factors among Iranian population, a few of them reporting from the central parts of Iran, examined the clustering of multiple risk factors in adult population [6, 7]. Based on the repots of the ministry of Health in Iran, Zanjan, is ranking as the fourth province of Iran for the rate of cardiovascular mortality. The aim of this study was to investigate the prevalence of the metabolic syndrome in a representative northern west Iranian urban population using the ATP-III guidelines.

## 2. Subjects and Methods

### 2.1. Study Population

This study analyzed data from a cross sectional study (Healthy Heart study), conducted by the Zanjan University of medical sciences between 2002 and 2003 in Zanjan, the capital of the Iranian province of the same name. The main ethnic groups living in the province are Azeries (Turks) and Kurds.

 In the original study the anthropometric parameters, nutritional status and cardiovascular risk factors in the population of Zanjan was assessed. A stratified, multistage random sampling was used and a total of 4000 subjects aged more than 15 years were enrolled in the study. We excluded 723 subjects for whom some information was missing and 336 people aged less than 20 years. The remaining subjects were 2941 subjects including 1396 men and 1545 women. Subjects completed a questionnaire which included dietary habits, past medical history, smoking status, physical activity, and educational levels.

All the subjects received oral information concerning the study and gave their written consent. The study was approved by the Ethical Committee of Zanjan University of medical sciences.

### 2.2. Measurements

In the original study Waist circumferences between the lowest rib and the iliac crest at the level of umbilicus were measured in duplicate to the nearest mm with flexible tape. 

Blood pressure was measured sitting with a random zero sphygmomanometer. Systolic (Korotkoff phase I) and diastolic (Korotkoff phase V) blood pressure was measured twice on the left upper arm and the average used for analysis. Average systolic blood pressure more than 130 mmHg or diastolic blood pressure more than 85 mmHg or current use of antihypertensive medications was defined as hypertension.

Laboratory measurements were done at the laboratory of Zanjan University of medical sciences, Valie-e-sasr Hospital. Plasma glucose was measured by the glucose-peroxidase colorimetric enzymatic method with a sensitivity of 5 mg/dl and intra-assay coefficients of variation (CV) I.7% in lower limit and 1.4% in upper limit concentrations. Inter-assay CV for the assay was 1.1% in lower limit and 0.6% in upper limit concentrations. Fasting plasma glucose (FPG) more than 100 mg/dl was defined abnormal in this study.

Serum Cholesterol and Triglyceride of all the participants were measured after 12–14 hours of fasting with colorimetric method with a sensitivity of 5 mg/dl. Intra-assay and inter-assay CV for the assay was 1.6% and 1.1% in lower limit and 0.6% and 0.9% for upper limit concentrations respectively. High-density lipoprotein cholesterol (HDL-C) was measured after precipitation of the apolipoprotein B-containing lipoproteins with phosphotungstic acid (Pars azmoon kit, Iran).

Hypertriglyceridemia was defined as triglyceride (TG) concentration more than 150 mg/dl. HDL Cholesterol less than 50 mg/dl in females and less than 40 mg/dl in males was considered to be abnormal.

In this study, subjects with three or more of the following five risk factors of the criteria of the modified NCEP III were defined as having metabolic syndrome: (1) triglycerides ≥150 mg/dl, (2) HDL cholesterol <40 mg/dl in men and <50 mg/dl in women, (3) systolic blood pressure ≥130 mmHg or diastolic blood pressure ≥85 mmHg, (4) fasting plasma glucose ≥100 mg/dl and (5) Truncal obesity (waist circumference more than 102 cm in men and >88 cm in women). Subjects with a history of hyperlipidemia, hypertension, or diabetes were considered to have the risk factor, regardless of the biochemical or clinical values.

### 2.3. Statistical Analyses

The data are presented as frequencies, percentages, and 95% confidence intervals. The prevalence of different abnormalities was compared using *x* ^2^ test. Logistic regression analysis was used to detect the value of variables such as body mass index (BMI) and hypercholesterolemia to predict the existence of metabolic syndrome. The analysis was done with SPSS 11.5 software package. *P* value of < .05 was considered statistically significant.

## 3. Results

The prevalence of the metabolic syndrome in the study population was 23.7% (CI 95%:22% to 25%, *P* : .001). The prevalence of metabolic syndrome was the same in women and men (24.4%, CI 95%: 22%–26% versus 23.1 %, CI 95%: 21%–25%, resp.) (*P* = .4). There was a significant age-related increase in the prevalence of metabolic syndrome in both of the genders. Prevalence of metabolic syndrome increased from 7.5% within the 20–29-year-old group to 44.7% in people more than 60 years of age. The prevalence of individual components of the metabolic syndrome is reported in Table 1. In men and women, respectively, central obesity was in 148 (10.6%) and 635 (41.4%), hypertension in 316 (22.6%) and 306 (19.8%), low HDL cholesterol in 880 (63%) and 1441 (93.3%), high triglycerides in 600 (43%) and 587 (38.4%), and impaired fasting glucose or diabetes in 252 (18.1%) and 296 (19.2%). Prevalence of other atherosclerosis risk factors in men and women, respectively, was diabetes in 65 (4.7%) and 88 (5.7%) and high total cholesterol ≥200 mg/dl in 428 (30.7%) and 613 (39.7%). Overweight (BMI: 25–29.9 kg/m^2^) was detected in 1068 (36.3%) and obesity (BMI => 30 kg/m^2^) was observed in 437 subjects (14.8%).

Low HDL-C was the most common metabolic abnormality in both sexes. Mean serum HDL-C was 36.9 ± 5.3 mg/dl in those with the metabolic syndrome and 46.7 ± 6 mg/dl in normal individuals without any risk factors (*P* < .001). Except for hypertension and hypertriglyceridemia, all abnormalities were more common in women than in men (*P* : .00). Most of those with metabolic syndrome had three components of the syndrome (75.6%), 170 subjects (24.4%) had four components. None of the people had five components.

Table 2 shows the mean value of coronary risk factors in subjects with metabolic syndrome as compared to those without. BMI ≥ 30 kg/m^2^ as well as high total and LDL cholesterol, which are not part of the metabolic syndrome, are also more prevalent among men and women with this condition. Elevated LDL-C more than 130 mg/dl was found in 13.5% of normal men versus 44.1% of those with metabolic syndrome (*P* : .00). In females high LDL-C was detected in 19.6% versus 58% in normal subjects and those with metabolic syndrome respectively (*P* : .00).

Within the 20–29-year-old group of the general population, 13.4% had no abnormality (23% of males and 5.6% of females), while this figure dropped to 3.4% in people above 60 years of age (5.8% of males and 0.9% of females). Totally 13.3% of males and only 3.4% of females had no cardiovascular risk factor in the population.

## 4. Discussion

The results of this study indicate that according to ATP III criteria, 23.7% of the studied adult population has metabolic syndrome. Since the study population is representative, the findings can be generalized to the whole urban population of the northern west of Iran.

The prevalence of MS varies considerably worldwide. Some of the differences in the prevalence of MS might arise from varied definitions of the syndrome. For instance, Trevisan et al. [8] reported a prevalence of 3–3.5% in Italy on the basis of the presence of all five criteria. However, a wide variation in the prevalence can be observed even with using the same diagnostic criteria. For example, the frequency of MS in a sample of the Chinese population was recorded as 9.8% for men and 17.8% for women [9]. In a rural area of South Korea, MS was found to affect 29.4% of the adult population above 40 years of age [10], and similar values were established in Mexico, where 26.6% of the population studied exhibited the syndrome [11].

 Although the prevalence of the metabolic syndrome in this study is higher than some previously reported from the USA, Italy, and Finland [4, 8, 10, 12], it is close to that reported from Brazil [13], Indian urban population [14], and some reports from the US [15, 16]. High prevalence of MS also has been reported from Tehran (33%) and Isfahan, two main capital cities in the central part of Iran (24.2%) [6, 7].

The exact reasons for high prevalence of MS in our study remain to be determined, but it is evident that substantial socioeconomic changes have occurred in the population over the past decades and the transition from a traditional to a western-like urban lifestyle has been associated with adverse changes in lifestyle habits. Based on the results of a national profile of non-communicable disease risk factors in Iran, 2005 [17] fiber intake is low in zanjan population (ninety four percent have less than three units per week of vegetables and fruits) and most of them use high saturated fat (67%) for daily cooking. Only 10% of the population has regular walking at least three times per week. Physical inactivity has been reported in 58.8% of males and 76.3% of females in Iran [17]. Based on international questionnaire for physical activity only 11% of Zanjan population have sever physical activity at least one time per week and 30% have moderate physical activity at least three times per week. Although high-fat, high-carbohydrate diet; and the sedentary lifestyle have been reported for Iranian population [18], the role of genetic factors should not be omitted. Although in Iran, women generally have less physical activity, and overweight and obesity are more common among them [19], we did not find a significant difference between men and women for the prevalence of MS. Ford and coworkers [4] also observed little overall difference between men and women (24% versus 23.4%, resp.); however, among African-Americans and Mexican-Americans the disorder was more common in women. In this study, the greatest difference observed between the two sexes was the prevalence of abdominal obesity (9.7% in men versus 38.3% in women). 

In our study the single most common abnormality was low HDL-C (overall 73%), which is more than what had previously been reported from USA [20], Turkey [21], Italy [22], Canada [23], and UK [24]. A high prevalence of low HDL-C has been reported previously in Iranian population [25, 26] and is very close to that was reported from Turkish [27]. This could not only be attributed to environmental factors but may also be due to genetic predispositions. Previous family and twin studies [28–30] have suggested that genetic polymorphism accounts for 40–60% of the interindividual variation in plasma HDL-C level. Surprisingly, low HDL-C was more prevalent in females in our study. Most of the women had HDL-C concentrations between 45 and 50 mg/dl and with lowering the cutoff points to 40 mg/dl the prevalence drops significantly in females (53% for women versus 63% for men).

A positive effect of age on the prevalence of the syndrome in both sexes was detected in this study and resulted in 45.5% of MS in subjects more than 50 years. This effect has been reported in other studies [4]. Age-related increases in insulin resistance have been shown in young, middle-aged, and elderly healthy normal-weight adults [31], and an age-related difference in the degree of clustering of risk variables [32] has been reported too.

In conclusion the present study from the northern west of Iran demonstrated that MS is a serious problem among the urban populations of this country affecting primarily older individuals. Since the Iranian population, composed mainly of those less than 30 years old, it is very likely that the prevalence of MS will be even greater in the next decades. The prevention and treatment of this condition is of major public concern and urgently requires the application of appropriate policies and considerable investment.

## Figures and Tables

**Table 1:** Prevalence of individual abnormalities of the metabolic syndrome by age in urban population of Zanjan, Iran. Numbers in parentheses are percent. FPG; fasting plasma glucose, TG; triglyceride, HDL; high density lipoprotein, WC; weist circumference High TG: TG > 150 mg/dl, Low HDL: HDL < 50 in female and <40 mg/dl in male, High WC: WC ≥ 88 cm in female and ≥102 cm in male, High BP: BP ≥ 130/85 mmHg.

Age groups (years)	FPG ≥ 100 mg/dl	High TG	Low HDL	High WC	High BP
Men (*n* : 1396)					
20–29	41 (9.9)	127 (30.4)	232 (55.5)	12 (2.9)	34 (8.1)
30–39	49 (14.8)	171 (52.3)	217 (65.6)	23 (6.9)	57 (17.2)
40–49	46 (17.1)	129 (47.8)	146 (65.2)	44 (16.3)	64 (23.7)
50–59	44 (29.3)	89 (58.6)	105 (69.1)	29 (19.1)	60 (39.5)
+60	72 (32.1)	83 (36.9)	150 (66.7)	114 (54)	101 (44.9)
Total	252 (18.1)	599 (43)	880 (63)	154 (9.7)	316 (22.6)
Women (*n* : 1545)					
20–29	33 (6.4)	119 (23)	482 (93)	79 (15.3)	22 (4.2)
30–39	60 (16.2)	124 (33.5)	346 (93.3)	142 (38.3)	33 (8.9)
40–49	59 (21.6)	133 (48.7)	258 (94.2)	159 (58)	59 (21.5)
50–59	60 (35.1)	102 (59.6)	157 (91.8)	114 (66.7)	76 (44.4)
+60	84 (40.2)	113 (54.1)	198 (93.8)	141 (66.8)	116 (55)
Total	296 (19.2)	591 (38.4)	1441 (93.3)	645 (38.3)	306 (19.8)

**Table 2:** Comparison between subjects with metabolic syndrome and normal people for the mean value of atherosclerosis risk factors. BMI, body mass index; WC, Waist circumference; BP, Blood pressure; LDL, low density lipoprotein; HDL, high density lipoprotein.

Variables	Men (*n* : 1396)			Women (*n* : 1545)		
	Normal* (*n* : 185)	Metabolic syndrome (*n* : 321)	*P* value	Normal (*n* : 52)	Metabolic syndrome (*n* : 376)	*P* value
BMI (kg/m^2^)	22.5 ± 3.8	26.4 ± 4.2	0.00	23 ± 4.2	28.5 ± 4.4	0.00
WC (Cm)	80.5 ± 10.7	94.2 ± 11	0.00	76.8 ± 9.8	93.2 ± 10.7	0.00
BP (mmHg)			0.00			0.00
Systolic	112 ± 9.4	140 ± 21.9		108.5 ± 9.8	139.7 ± 23	
Diastolic	73.2 ± 8.1	86.2 ± 12		72.3 ± 8.4	87 ± 12.7	
LDL-C (mg/dl)	99.3 ± 30.5	123.6 ± 37.7	0.00	105.4 ± 32	137 ± 42	0.00
Cholesterol (mg/dl)	162 ± 30	203 ± 40	0.00	175.3 ± 33.6	218 ± 50	0.00
HDL-C (mg/dl)	44.5 ± 4.4	34.5 ± 4.4	0.00	54.2 ± 5.9	37.1 ± 5.6	0.00
Triglyceride (mg/dl)	91 ± 29.7	260 ± 141	0.00	82 ± 31	247 ± 136	0.00

*Normal people are those without any components of metabolic syndrome.
